# Hyperammonemia in a pregnant woman with citrullinemia type I: a case report and literature review

**DOI:** 10.1186/s12884-022-05298-3

**Published:** 2022-12-19

**Authors:** Yimeng Zhou, Xiaoguang Dou, Chong Zhang, Rong He, Yang Ding

**Affiliations:** 1 Department of Infectious Diseases, Shengjing Hospital of China Medical University, Sanhao Street No.36, Heping District, Shenyang, 110004 China; 2 Department of Clinical Genetics, Shengjing Hospital of China Medical University, Sanhao Street No.36, Heping District, Shenyang, 110004 China

**Keywords:** Citrullinemia, Hyperammonemia, Pregnancy, Urea cycle disorders, Case report

## Abstract

**Background:**

Citrullinemia type I (CTLN1) is a rare urea cycle disorder (UCD) with few adult cases described so far. Diagnosis of late-onset CTLN1 is difficult, and delayed treatment may increase the risk of severe hyperammonemia. Pregnancy is an important risk factor for women with CTLN1. However, the clinical manifestations of CTLN1 in a pregnant woman may be mistaken for pregnancy side effects and ultimately delay a timely diagnosis.

**Case presentation:**

A 34-year-old woman developed vomiting and disturbance of consciousness after 12 weeks of gestation. A blood test showed hyperammonemia (454 μg/dL) with normal liver function tests. She fell into a deep coma, and her serum ammonia level increased to 800 μg/dL. Continuous renal replacement therapy (CRRT) was administered as a diagnostic treatment for UCD and serum ammonia. This patient’s case was complicated by co-infection; her dependents decided to withdraw life support and the patient died. She was diagnosed with CTLN1 by analyses of plasma amino acids, urinary orotic acid, and second-generation gene sequencing.

**Discussion and conclusion:**

When a patient displays symptoms of emesis and disturbance of consciousness in early pregnancy, blood ammonia should be monitored, and UCD should be considered, particularly for patients with hyperammonemia in the absence of severe liver function abnormalities.

## Background

Ammonia arises from the breakdown of proteins and is produced mainly in the intestines by gut bacteria; it can also be produced in the kidneys and muscles [1, 2]. Ammonia crosses the blood-brain barrier readily and, at increased concentrations, is toxic to the brain. The detoxification of ammonia, a metabolite of protein catabolism, is through the synthesis of glutamine and urea in the liver, which are eventually excreted in the urine. As a result, plasma concentrations of ammonia in the systemic circulation are very low (< 40 mmol/L) in healthy adults. Hyperammonemia develops in patients with liver disease or if the urea cycle cannot control the ammonia load [3, 4]. Most adult cases of hyperammonemia are complicated by severe liver disease, other causes are much less common [5]. Inherited urea cycle defects (UCDs) can lead to hyperammonemia, which causes brain damage. UCDs are caused by inherited deficiencies of one of the six enzymes in the urea cycle [6]. Citrullinemia type I (CTLN1) is a UCD caused by decreased activity of argininosuccinate synthase 1, a critical urea cycle enzyme. Typically, classical citrullinemia leads to hyperammonemia in the newborn period and early death occurs if untreated. However, in some patients, symptomatic hyperammonemia develops during childhood, adulthood, or during/shortly after pregnancy, referred to as the late-onset form [7, 8].

We herein report the case of a pregnant woman with CTLN1 who vomited and went into a coma.

## Case presentation

A healthy 34-year-old female was admitted to hospital after 12 weeks of gestation. The patient began to have symptoms of nausea and vomiting at 5 weeks of pregnancy. The symptoms worsened over a 3-day period, and she was admitted to hospital for inability to eat. The patient had no previous liver disease or other diseases. The patient denied a history of drinking alcohol, and she had no recent history of medication use that could potentially be toxic to the liver. Her parents were not consanguineous.

When admitted to the hospital, the patient had a routine blood examination which included tests for liver function, kidney function, potassium, sodium, chloride, and glucose, all of which were within the normal range. Prothrombin time (PT) was extended by 3 seconds, and C-reactive protein was slightly elevated. Hepatitis virus markers were negative. The patient had an abdominal ultrasound examination of the fetal sac, fetal bud, and fetal heart, all of which were normal. Initially, the patient was given fluid replacement and vitamin B_6_ and B_1_ supplements.

**Fig. 1:**
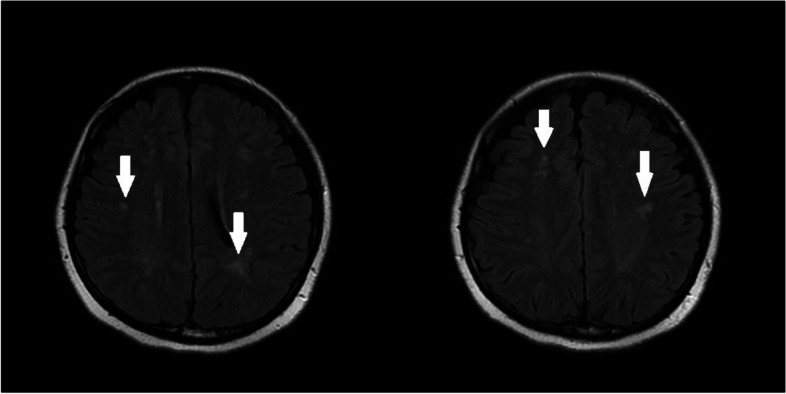
On the third day of admission, MRI of the head showed scattered intraluminal infarcts or demyelinating lesions in the brain

On the second day of admission, the patient had no emesis but did not cooperate with the medical staff’s questions or medical examinations. She had difficulty falling asleep, and was given a 10-mg intramuscular injection of diazepam after notification of the risk. On the morning of the third day of admission, a magnetic resonance imaging (MRI) study of the head was performed. The result showed scattered intraluminal infarcts or demyelinating lesions in the brain (Fig. 1). She experienced sudden convulsions and showed no response to attempts at interaction by staff during the night of the third day of admission. She was given oxygen by mask, a slow push of diazepam, and quick intravenous drip of magnesium sulfate. She was transferred to the intensive care unit (ICU) and was given cefuroxime; medications for phlegm reduction, convulsions, and hypertension; and sedative treatment.

**Fig. 2:**
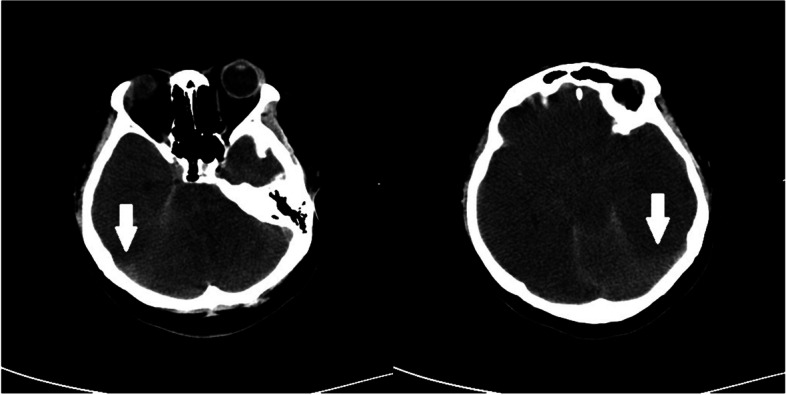
On the fifth day of admission,CT of the head showed that diffuse cerebral edema

**Fig. 3:**
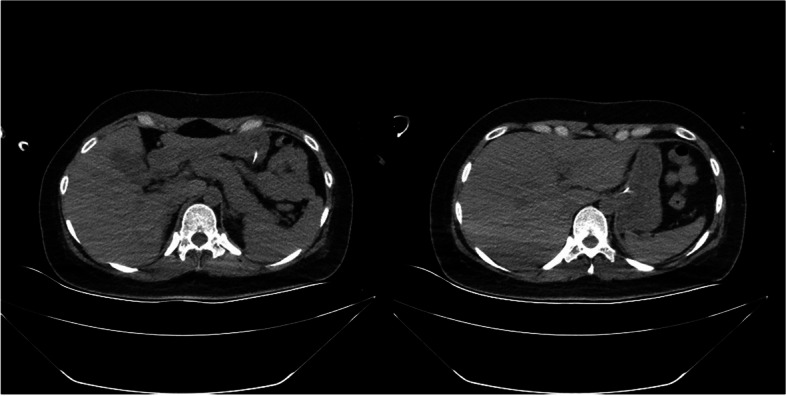
Abdominal CT on the fifth day of admission.Both the liver and spleen were normal in size and shape

When transferred to ICU, blood examination revealed a leukocyte level of 20.8 × 10^9^/L, neutrophils 89.6%, slightly elevated serum chloride, PT of 21.5 seconds, plasma thromboplastin antecedent of 42%, and plasma ammonia of 503 μmol/L; hemoglobin, platelet level, liver function transaminases, bilirubin, kidney function, and potassium and sodium levels were all within normal limits. Antineutrophil cytoplasmic antibody, anticardiolipin antibody, and HLA-B27 were negative on blood tests performed. On the fifth day of admission, A computed tomography (CT) of the brain was performed. The CT report showed diffuse cerebral edema and a small amount of subarachnoid hemorrhage (Fig. 2). Multiple areas of inflammation and exudative lesions were noted in both lungs, along with consolidation of the left lower lobe. There was bilateral pleural effusion. The liver and spleen were normal (Fig. 3).

**Fig. 4:**
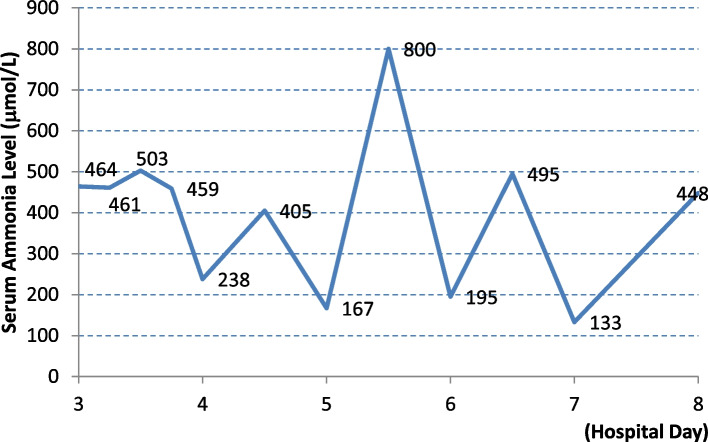
Serum ammonia level significantly increased every time

After consultation with all the relevant departments, combined with nausea and vomiting symptoms, high plasma ammonia, convulsions and mental symptoms, this was considered as urea cycle disorder. She was given plasma exchange and continuous renal replacement therapy (CRRT) treatment. She was also administered lactulose combined with vinegar saline enema and other supportive treatments, tracheal intubation to ensure airway patency. Because the patient had hyperchloric acidosis, arginine was not given. Plasma ammonia significantly increased every time it was tested (Fig. 4). On the seventh day of admission, antibiotics were adjusted and the patient received continued CRRT and plasma exchange treatment, along with levocarnitine to remove toxins from the body. On the eighth day of admission, the patient was still in a coma and had no spontaneous breathing. Glasgow Coma Scale score was 3 and there was intermittent high fever. After careful consideration, her family members decided to discontinue plasma exchange and CRRT, and requested removal of mechanical ventilation and all medication therapy. The patient was pronounced dead that night.

The result of urinary organic acids came out a few days later, suggested that the levels of many organic acids such as uracil and various orotic acids were high. Analysis of blood amino acids showed higher citrulline 541.57 (normal range, 4-35), ornithine 214.94 (normal range, 10-120), and aspartic acid 108.53 (normal range, 10-100) levels, indicating a UCD.

### Molecular investigation

The patient’s whole blood was collected for high-throughput sequencing; the patient’s family members refused to have their blood tested. The analysis showed that there were two heterozygous variations in the argininosuccinate synthase 1, or *ASS1*, gene of the patient. The first variation was c.421-2A > G, causing the change of amino acid; this was a splicing variation. According to the Criteria for Classifying Pathogenic Variants from the American College of Medical Genetics and Genomics (ACMG) guidelines, this variation was initially determined to be pathogenic (PVS1, a zero-effect mutation that may lead to loss of gene function). The Human Gene Mutation Database (HGMD) database already had a related report of citrullinemia for this site (PS1) [9]. The pathogenicity of this site in the ClinVar database was pathogenic (CTLN1).

The second variation was c.1046 T > G (variation of nucleotide 1046 in the coding region from thymine to guanine) causing the change of amino acid p.V349G (valine to glycine at amino acid 349), which is a missense mutation.

The final diagnosis was CTLN1.

## Discussion

Although UCD onset often occurs in pediatric-age patients, there is considerable evidence for late-onset cases recognized in adulthood [10]. CTLN1 presents as a spectrum, including a neonatal acute form, a milder late-onset form, and a form in which women have onset of the disease during pregnancy or postpartum without symptoms or hyperammonemia [11]. Symptoms of UCDs may include hypotonia, emesis, lethargy, seizures, coma, ataxia, anorexia, abnormal behavioral patterns, dysarthria, weakness, and dementia. The clinical signs and symptoms of late-onset UCDs may be subtle, unspecific, and episodic [12]. In our case, the patient did not show obvious symptoms of discomfort. Her family members said that she had a history of a bad temper. She was a technician in the radiology department of a hospital, and she could work normally. She was admitted to the hospital under our care at 12 weeks of pregnancy. She had emotional and behavioral abnormalities that began after becoming pregnant. The clinical features of our patient included nausea and vomiting, psychomotor agitation, anxiety, behavior and mood changes, and neurologic symptoms. Gastrointestinal symptoms could easily be considered as side effects of pregnancy, and emotional abnormalities might be mistaken for prenatal anxiety. Hence, we failed to identify UCD early. The plasma ammonia level was significantly higher; this, combined with the clinical manifestations, led to the conclusion that this patient had a UCD.

Heterozygous women of UCDs may be asymptomatic or with varying degree of clinical manifestation. The severity of the disease depends on the degree of inactivation of the mutated X-chromosome [13]. Nutritional changes may contribute to nitrogen imbalance and precipitate metabolic decompensation and presentation of UCDs. Hyperemesis gravidarum is a risk factor for metabolic decompensation due to caloric deficit. This may lead to hyperammonemia. Commonly metabolic decompensation occurs in postpartum period. Involution of the uterus is associated with tissue catabolism due to collagen breakdown. This may cause overload of the capacity of urea cycle. Caesarean section, birth trauma, and infection may lead to additional catabolic stress. Blood transfusion may represent an increase of protein load [14, 15]. Our patient was usually asymptomatic, which might be related to the degree of X-chromosome inactivation. Unfortutely,her parents refused high-throughput sequencing. The onset in early pregnancy was precisely due to hyperemesis gravidarum and long-term fasting. This might cause nitrogen imbalance and the onset of hyperammonemia.

Several cases of CTLN1 in pregnant women have been reported in the past. Potter et al. [16] reported a woman diagnosed with CTLN1 at birth. She had no obvious symptoms of discomfort in daily life. Häberle et al. [17] reported three women who presented in hyperammonemic coma shortly after delivery. One died and two survived without neurologic sequelae. They were eventually diagnosed with CTLN1. A healthy 28-year-old pregnant woman at 22 weeks of pregnancy presented with altered mental status, encephalopathy, and abnormal liver tests. Her ammonia level was 208 μg/dL. Eventually, she was found to have CTLN1. The patient began a low protein diet along with arginine, carnitine, and sodium phenylbutyrate. The patient’s mental status normalized, and she had an uncomplicated term delivery of a healthy male baby [5]. Salek et al [18] also reported a 25-year-old woman at 19 weeks of pregnancy with symptoms of nausea, emesis, and edema of both lower limbs in 2010. Elevated aminotransferases, jaundice, an elevated international normalized ratio, and confusion are typical findings of idiopathic acute liver failure. CTLN1 was confirmed by molecular testing. Her ammonia levels remained low, and she delivered a male infant who appeared completely normal at birth and at 2 months of age.

CTLN1 is inherited in an autosomal recessive manner and is due to a heterogeneous group of variations in the *ASS1* gene on chromosome 9. The *ASS1* gene consists of 16 exons encoding 412 amino acids. A wide range of variations have been implicated in CTLN1. The pathogenic variants p.Gly390Arg, p.Arg363Trp, and p. Gly14Ser have been identified with a severe phenotype. In contrast, p.Tyr190Asp, p.Trp179Arg, p.Val263Met, and p.Val269Met were already described in patients with the late-onset form of the disease [19, 20].

The diagnosis of CTLN1 is established in a proband with elevated plasma ammonia concentration (> 150 μmol/L; may range up to ≥2000-3000 μmol/L) and plasma citrulline concentration (usually > 1000 μmol/L), and/or by the identification of biallelic pathogenic variants in *ASS1* on molecular genetic testing [11]. Our patient was admitted to hospital because of vomiting. She was supplemented with vitamin B_1_ to prevent Wernicke encephalopathy. After this, neurologic symptoms appeared. We considered that it might be autoimmune encephalopathy. But on laboratory testing, the level of plasma ammonia was significantly higher. This, combined with no previous history of liver disease, led us to consider a UCD. Therefore, patients with a UCD must be diagnosed correctly based on a review of the patient’s history and the laboratory and imaging findings. However, analyses of amino acids can take a few days. We didn’t know exactly what type of UCD this patient had.

In the past, 20 metabolic centers in five European countries shared their experience with patients presenting with non-classical UCD, including data from 208 patients with non-classical UCD and deficiencies of ornithine transcarbamylase (OTC; 58%), argininosuccinate synthetase (20%), argininosuccinate lyase (15%), arginase 1 (4%), carbamoyl phosphate synthetase 1 (1%), N-acetylglutamate synthase (1%), and the hyperornithinemia-hyperammonemia-homocitrullinuria syndrome (1%) [21]. We initially considered that this patient might have OTC deficiency because OTC deficiency accounts for the majority of adult patients with UCD who present with acute encephalopathy and is the most common form of UCD [3]. Citrin deficiency is due to a variation of SLC25A13 which encodes for citrin, an aspartate-glutamate carrier that transports glutamate into and aspartate out of mitochondria. The clinical course in adults with citrin deficiency is milder than that of CTLN1 [22]. For others diagnostically, organic acidaemias (OAs) must be differentiated from UCDs. The presence of metabolic acidosis and ketonuria suggests OA in neonates, whereas respiratory alkalosis is often seen in UCDs [23].

Acute hyperammonemia in patients with UCD may progress rapidly. The aims for treatment are to reduce production of nitrogenous waste and to lower plasma ammonia levels quickly. Dialysis should be performed immediately, and nitrogen scavengers (sodium benzoate and sodium phenylacetate) and arginine hydrochloride should be given. Early in the treatment, dialysis plays a significant role in rapid ammonia removal. Furthermore, dialytic interventions through acute dialysis in patients with hyperammonemia lead to improved outcomes [6, 24]. Carnitine is also useful for reducing plasma ammonia levels in patients with acute UCD by activating carbamoylphosphate synthase 1 and increasing the elimination of free radicals by participating in the transportation of short-chain fatty acids. Treatment with L-carnitine significantly reduced blood ammonia and improved patient mental status [2, 25]. For the treatment of chronic citrullinemia, patients should have a restricted protein diet and take sodium phenylbutyrate or glycerol phenylbutyrate orally. There is also a case report of CTLN1 for whom follow-up was performed with carglumic acid treatment over a long-term period. Liver transplantation is the only known cure for CTLN1 [26]. After considering the hyperammonemia in this patient to be the result of a UCD, we applied blood purification treatment and gave levocarnitine to remove toxins. Because of the presence of hyperchloric acidosis, the use of arginine was contraindicated, and no arginine treatment was given. Unfortunately, the patient’s co-infection aggravated her disease. The infection was fatal to the pregnancy and worsened the hyperammonemia. The family members decided to withdraw treatment after comprehensive consideration, and the patient died.

## Conclusion

This report aims to raise clinical awareness in considering mental abnormalities as a presenting symptom of UCDs in pregnant women. Nausea and vomiting are easily overlooked as accompanying symptoms in early pregnancy. When associated with behavioral symptoms, they need to be taken seriously. These patients can develop acute hyperammonemic encephalopathy incidentally by various triggers in adulthood, especially pregnancy. When we encounter patients with unusual hyperammonemia, CTNL1 should be considered so that fatal outcomes can be avoided through prompt diagnosis and adequate treatment. Molecular analysis is the method of choice in making a differential UCD diagnosis.

## Data Availability

The datasets used and/or analysed during the current study are available from the corresponding author on reasonable request.

## Abbreviations

CTLN1: Citrullinemia type I
UCD: Urea cycle disorder
CRRT: Continuous renal replacement therapy
PT: Prothrombin time
MRI: Magnetic resonance imaging
ICU: Intensive care unit
CT: Computed tomography
ACMG: American College of Medical Genetics and Genomics
HGMD: Human Gene Mutation Database
OTC: Ornithine transcarbamylase
OA: Organic acidaemia
